# Supplementary material for: Time-calibrated phylogenetic and chromosomal mobilome analyses of Staphylococcus aureus CC398 reveal geographical and host-related evolution
Source: Nat Commun. 2024 Jul 1;15:5526. doi: 10.1038/s41467-024-49644-9 (PMC11217367; doi:10.1038/s41467-024-49644-9)
Supplement: Supplementary file 1 — Supplementary Information [file 41467_2024_49644_MOESM1_ESM.pdf]

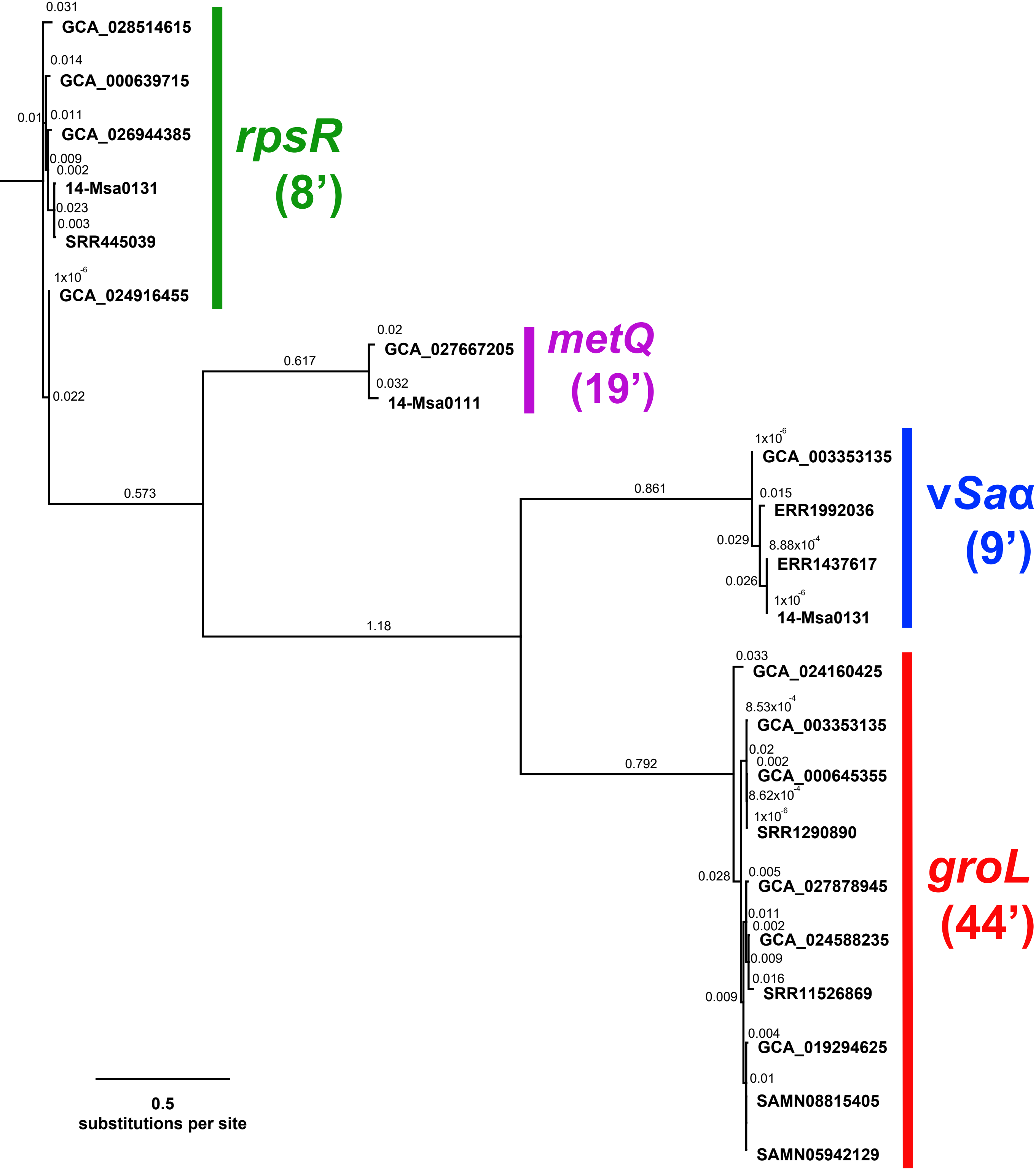

Supp. Fig. 1: SaGIs integrase families.

Phylogenetic tree based on a multiple sequence alignment of the nucleotide sequence of integrases from SaGIs integrating at four different chromosomal positions. Distance expressed in substitutions per site.

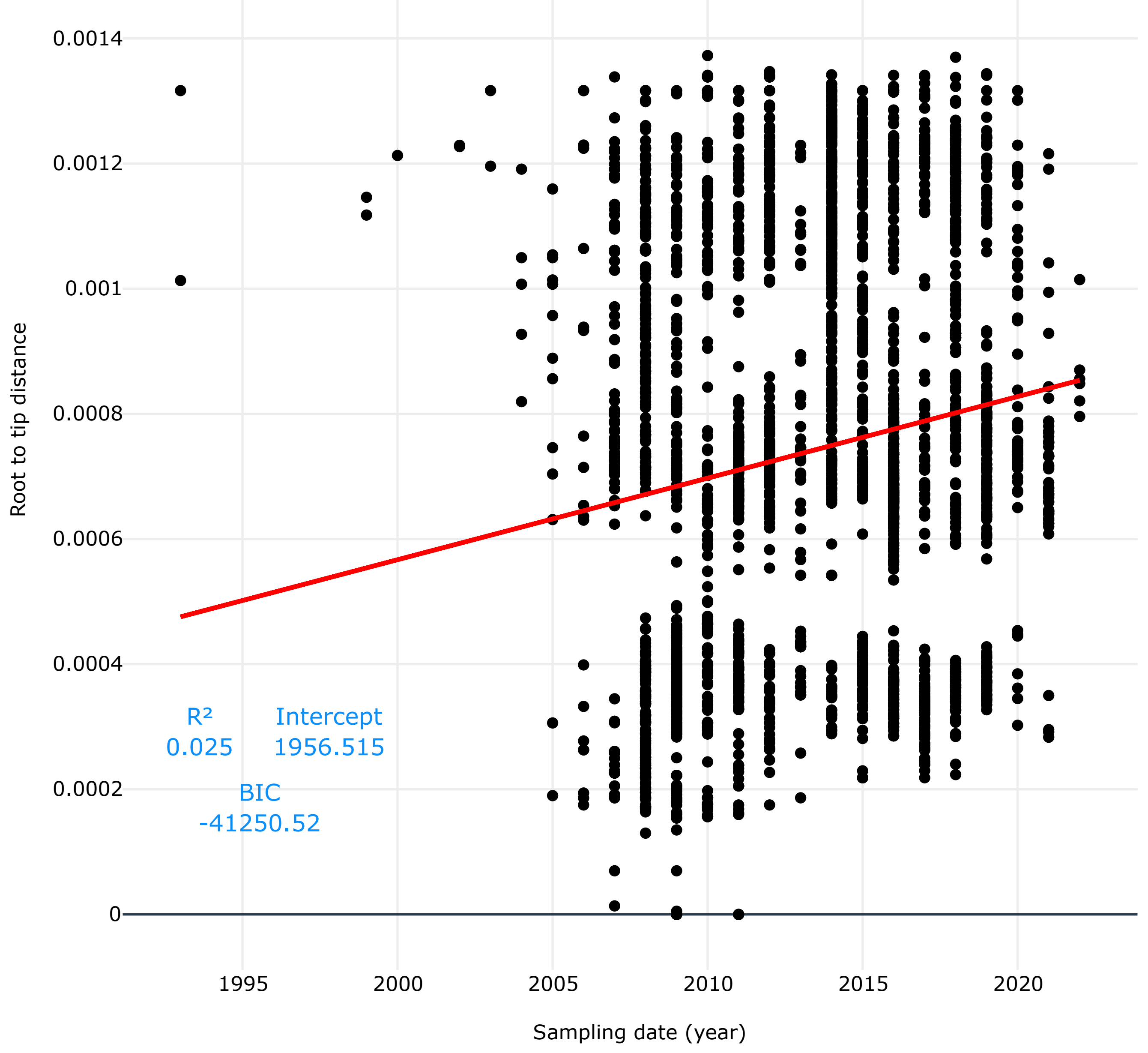

Supp. Fig. 2: Exploratory analysis and determination of time-signal in the cgSNP data of CC398. Root to tip regression analysis against sampling date for the entire collection of *S. aureus* CC398. **BIC**: Bayesian Information Criterion.

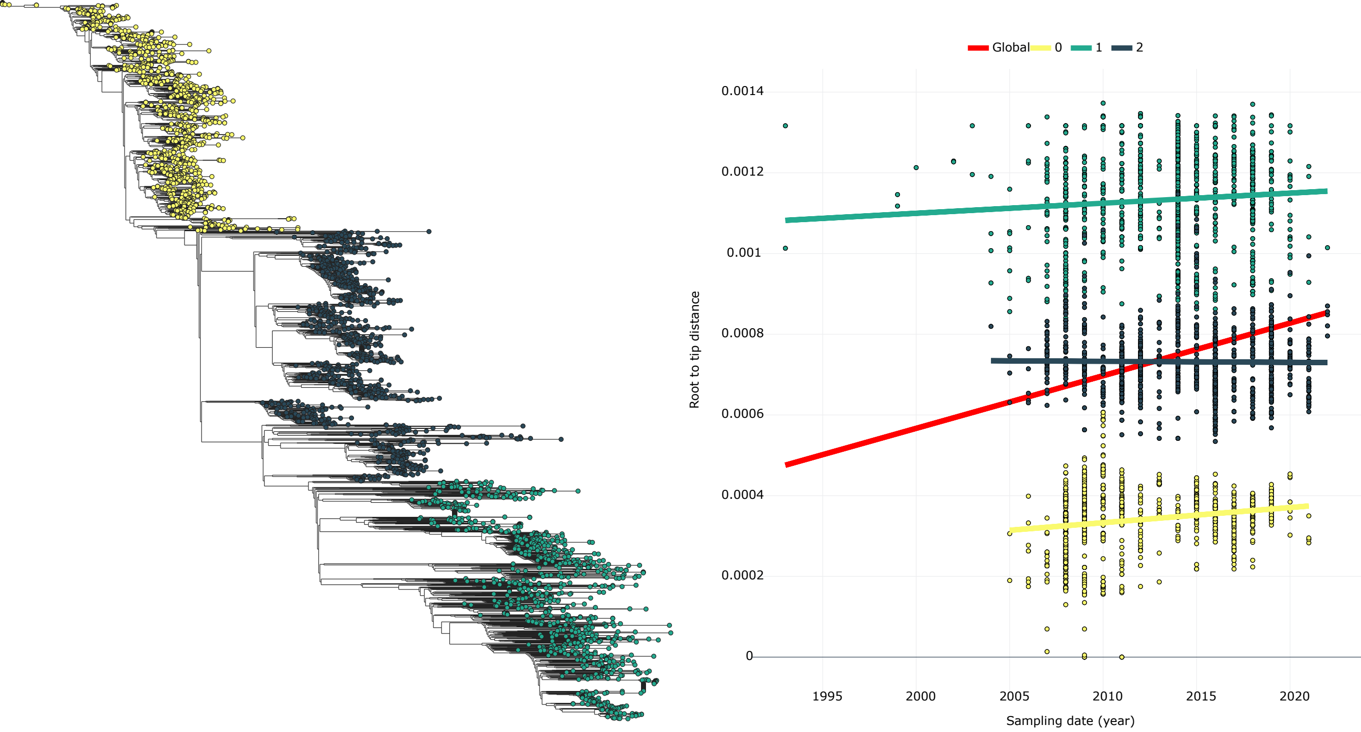

Supp. Fig. 3: Root to tip analysis of the obtained putative local clocks. Parameters used were 3 clocks and a minimum of 500 isolates per group for the search.
