# Supplementary material for: Time-calibrated phylogenetic and chromosomal mobilome analyses of Staphylococcus aureus CC398 reveal geographical and host-related evolution
Source: Nat Commun. 2024 Jul 1;15:5526. doi: 10.1038/s41467-024-49644-9 (PMC11217367; doi:10.1038/s41467-024-49644-9)
Supplement: Supplementary file 3 — Description of Additional Supplementary Files [file 41467_2024_49644_MOESM3_ESM.pdf]

<u>Description of Additional Supplementary Files</u>

Supplementary Data 1: Metadata, AMR and partial virulence profile associated with the 3128 <i>S. aureus</i> CC398 analyzed in the study.
