# Supplementary material for: Time-calibrated phylogenetic and chromosomal mobilome analyses of Staphylococcus aureus CC398 reveal geographical and host-related evolution
Source: Nat Commun. 2024 Jul 1;15:5526. doi: 10.1038/s41467-024-49644-9 (PMC11217367; doi:10.1038/s41467-024-49644-9)
Supplement: Supplementary file 5 — Reporting Summary [file 41467_2024_49644_MOESM5_ESM.pdf]

nature portfolio

Corresponding author(s): Prof. Dr. Vincent Perreten

Last updated by author(s): May 23, 2024

Reporting Summary

Nature Portfolio wishes to improve the reproducibility of the work that we publish. This form provides structure for consistency and transparency in reporting. For further information on Nature Portfolio policies, see our Editorial Policies and the Editorial Policy Checklist.

Statistics

For all statistical analyses, confirm that the following items are present in the figure legend, table legend, main text, or Methods section.

n/a	Confirmed	
☐	☒	The exact sample size (*n*) for each experimental group/condition, given as a discrete number and unit of measurement
☒	☐	A statement on whether measurements were taken from distinct samples or whether the same sample was measured repeatedly
☒	☐	The statistical test(s) used AND whether they are one- or two-sided<br>*Only common tests should be described solely by name; describe more complex techniques in the Methods section.*
☒	☐	A description of all covariates tested
☒	☐	A description of any assumptions or corrections, such as tests of normality and adjustment for multiple comparisons
☐	☒	A full description of the statistical parameters including central tendency (e.g. means) or other basic estimates (e.g. regression coefficient) AND variation (e.g. standard deviation) or associated estimates of uncertainty (e.g. confidence intervals)
☒	☐	For null hypothesis testing, the test statistic (e.g. *F*, *t*, *r*) with confidence intervals, effect sizes, degrees of freedom and *P* value noted<br>*Give P values as exact values whenever suitable.*
☐	☒	For Bayesian analysis, information on the choice of priors and Markov chain Monte Carlo settings
☒	☐	For hierarchical and complex designs, identification of the appropriate level for tests and full reporting of outcomes
☒	☐	Estimates of effect sizes (e.g. Cohen's *d*, Pearson's *r*), indicating how they were calculated

Our web collection on statistics for biologists contains articles on many of the points above.

Software and code

Policy information about availability of computer code

Data collection	To download sequencing reads from the SRA we used fasterq-dump version 3.0.1, for genome assemblies we used the command line E-utility functions of Entrez.
Data analysis	All tools and packages used for this analysis are publicly available and fully described in the “Methods” section.

For manuscripts utilizing custom algorithms or software that are central to the research but not yet described in published literature, software must be made available to editors and reviewers. We strongly encourage code deposition in a community repository (e.g. GitHub). See the Nature Portfolio guidelines for submitting code & software for further information.

Data

Policy information about availability of data

All manuscripts must include a data availability statement. This statement should provide the following information, where applicable:

- Accession codes, unique identifiers, or web links for publicly available datasets
- A description of any restrictions on data availability
- For clinical datasets or third party data, please ensure that the statement adheres to our policy

Accession numbers of all the strains used in the study are available in the Supplementary Data 1.

Research involving human participants, their data, or biological material

Policy information about studies with human participants or human data. See also policy information about sex, gender (identity/presentation), and sexual orientation and race, ethnicity and racism.

Reporting on sex and gender	n/a
Reporting on race, ethnicity, or other socially relevant groupings	n/a
Population characteristics	n/a
Recruitment	n/a
Ethics oversight	n/a

Note that full information on the approval of the study protocol must also be provided in the manuscript.

Field-specific reporting

Please select the one below that is the best fit for your research. If you are not sure, read the appropriate sections before making your selection.

☐ Life sciences ☐ Behavioural & social sciences ☒ Ecological, evolutionary & environmental sciences

For a reference copy of the document with all sections, see nature.com/documents/nr-reporting-summary-flat.pdf

Ecological, evolutionary & environmental sciences study design

All studies must disclose on these points even when the disclosure is negative.

Study description	Time-scaled evolutionary analysis, antimicrobial resistance, virulence and mobile genetic elements profiling of ***Staphylococcus aureus*** Clonal Complex 398
Research sample	A total of 3128 genome sequences, including 136 ***S. aureus*** CC398 strains from Switzerland and 2992 international strains.
Sampling strategy	All available (from the best of our knowledge) sequence data from projects involving MRSA CC398 was retrieved from the NCBI GenBank. For regions where MRSA was absent or less prevalent, MSSA CC398 were retrieved.
Data collection	*Describe the data collection procedure, including who recorded the data and how.*
Timing and spatial scale	At August 2023, all available (from the best of our knowledge) relevant sequence data was retrieved from the NCBI GenBank.
Data exclusions	No MRSA CC398 available was purposefully excluded.
Reproducibility	All analysis were repeated at least twice and all attempts (even with different parameter adaptations) were successful.
Randomization	n/a
Blinding	n/a

Did the study involve field work? ☐ Yes ☒ No

Reporting for specific materials, systems and methods

We require information from authors about some types of materials, experimental systems and methods used in many studies. Here, indicate whether each material, system or method listed is relevant to your study. If you are not sure if a list item applies to your research, read the appropriate section before selecting a response.

Materials & experimental systems

n/a	Involved in the study
☒	☐ Antibodies
☒	☐ Eukaryotic cell lines
☒	☐ Palaeontology and archaeology
☒	☐ Animals and other organisms
☒	☐ Clinical data
☒	☐ Dual use research of concern
☒	☐ Plants

Methods

n/a	Involved in the study
☒	☐ ChIP-seq
☒	☐ Flow cytometry
☒	☐ MRI-based neuroimaging

Plants

Seed stocks	*n/a*
Novel plant genotypes	*n/a*
Authentication	*n/a*
